# The Validation and Assessment of Machine Learning: A Game of Prediction from High-Dimensional Data

**DOI:** 10.1371/journal.pone.0006287

**Published:** 2009-08-04

**Authors:** Tune H. Pers, Anders Albrechtsen, Claus Holst, Thorkild I. A. Sørensen, Thomas A. Gerds

**Affiliations:** 1 Tune H. Pers Center for Biological Sequence Analysis, Department of Systems Biology, The Technical University of Denmark, Kongens Lyngby, Denmark; 2 Anders Albrechtsen Department of Biostatistics, University of Copenhagen, Copenhagen, Denmark; 3 Claus Holst Institute of Preventive Medicine, Copenhagen University Hospitals, Center for Health and Society, Copenhagen, Denmark; 4 Thorkild I. A. Sørensen Institute of Preventive Medicine, Copenhagen University Hospitals, Center for Health and Society, Copenhagen, Denmark; 5 Thomas A. Gerds Department of Biostatistics, University of Copenhagen, Copenhagen, Denmark; University of Swansea, United Kingdom

## Abstract

In applied statistics, tools from machine learning are popular for analyzing complex and high-dimensional data. However, few theoretical results are available that could guide to the appropriate machine learning tool in a new application. Initial development of an overall strategy thus often implies that multiple methods are tested and compared on the same set of data. This is particularly difficult in situations that are prone to over-fitting where the number of subjects is low compared to the number of potential predictors. The article presents a game which provides some grounds for conducting a fair model comparison. Each player selects a modeling strategy for predicting individual response from potential predictors. A strictly proper scoring rule, bootstrap cross-validation, and a set of rules are used to make the results obtained with different strategies comparable. To illustrate the ideas, the game is applied to data from the Nugenob Study where the aim is to predict the fat oxidation capacity based on conventional factors and high-dimensional metabolomics data. Three players have chosen to use support vector machines, LASSO, and random forests, respectively.

## Introduction

A researcher faced with complex data often needs a strategy to investigate the relationship between predictor variables and response. Classical methods like maximum likelihood cannot be applied if the data is high-dimensional in the sense that the number of predictor variables by far exceeds the number of subjects in the study. Machine learning tools are more generally available and have proven successful in a variety of studies [1], but they are typically not tailored to the specific problem at hand. This complicates the choice between different machine learning tools, and had the problem and the data been given to another researcher, most likely the strategy and potentially also the results would have been different. For conclusion making it is thus crucial to be able to assess differences between the results obtained with different strategies for the same research question.

Machine learning tools are automated approaches which combine variable selection and regression analysis [2]. Most machine learning tools are designed for prediction and usually they do not quantify the associations of the involved variables with p-values and confidence intervals. A strength, which is common to many machine learning tools, is their applicability when the number of subjects is considerably lower than the number of predictor variables. The practical value of the resulting models, however, is often unclear, in particular when the tool is applied by someone who is untutored in its niceties [3]. Most methods have tuning parameters to optimize the results. For example, classical stepwise elimination uses a threshold for the p-value of variables to be included in the next step of the algorithm. A second example is the random forest approach [4] where the model builder can vary the number of decision trees and the fraction of variables tried at each split of the single trees. Given the large variety of available tools, model and tuning steps, it is clear that the results of a given application depend on the model builder's preferences, dedication, and experience.

In many areas of applied statistics it still is common practice to develop the model building strategy during the data analysis, and then to treat the finally selected model as if it was known in advance. This has been criticized for example in [5]. More generally, any data dependent optimization of the model selection procedure can have a considerable impact on the final model, and may also lead to useless models and wrong conclusions [6]. This has to be considered carefully when a model is evaluated. Ideally all models should be compared by means of their performance on a large independent validation sample. However, independent data from the same population are not generally available, and even if they are, then one could merge them with the existing data to enhance the sample size. Internal model validation is therefore an essential part of model building [7].

In this article we present the VAML (Validation and Assessment of Machine Learning) game. The game aims at building a model for individual predictions based on complex data. The game starts by electing a referee who samples a reasonable number of bootstrap subsets or subsamples from the available data. Each player chooses a strategy for building a prediction model. The referee shares out the bootstrap samples and the players apply their strategies and build a prediction model separately in each bootstrap sample. The referee then uses the data not sampled in the respective bootstrap steps and a strictly proper scoring rule [8]–[10] to evaluate the predictive performance of the different models. This procedure is called bootstrap-cross-validation [11]–[15]. For the interpretation of the results it is most important that all modeling steps are repeated in each bootstrap sample and that the same set of bootstrap samples is used for all strategies. These insights are formulated as fixed rules of the game.

For the purpose of illustrating the VAML game, we applied it to metabolomics data collected on subjects from the multi-center Nugenob study (www.nugenob.org). For 99 subjects we considered 8525 potential predictor variables consisting of anthropometric measures and high-dimensional metabolomic profiles from blood plasma obtained by nuclear magnetic resonance (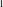
H-NMR) and liquid chromatography mass spectrometry (LC-MS) techniques. The aim of the game was to predict the fat oxidation capacity measured by the respiratory quotient. Active players were the first two and the last author of this work, who chose the following strategies for building prediction models: random forests regression [4], support vector machines (SVMs) [16], and LASSO [17]. Each players strategy was then adapted to build models for predicting the subject specific probability distribution of the respiratory quotient. The criterion for winning the game was the prediction error defined by the expected value of the continuous rank probability score [10] for continuous outcomes. The estimation of the prediction performance was based on bootstrap-cross-validation, where 100 bootstrap samples of size 80 were drawn without replacement for building the models and the remaining 19 subjects were used for internal validation.

## The VAML game

### Material

A VAML game requires measurements of a 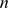-dimensional response vector 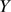 and a 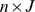 predictor matrix 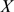 containing the values for 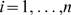 subjects and 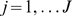 variables. We use the notation 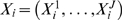. For the standard form of the game, the response is either a single continuous variable, a binary variable, or a right censored event time. The predictor matrix consists of subject specific information of any kind, and may include a mixture of behavioral factors, genotype, conventional factors, like gender and age, and environmental variables.

### Aim

The aim is to build a prediction model for the conditional probability distribution of the response variable given the predictor matrix. The finally selected prediction model should assign to each (new) subject a probabilistic prediction for the potential values of the response variable based on the subjects predictor values. For example, if the response is a survival time, then the model predicts a survival probability for each time point in the range of the survival distribution.

### Choosing a method

The players derive strategies for selecting a prediction model. Often it will be advisable to rely on an approved method for data analysis. Generally methods are called unsupervised if the prediction model depends only on the predictor matrix of the sample and is independent of the corresponding response values 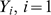, 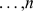. Principal component analysis is an example of an unsupervised method. Supervised methods on the other hand select a model by using the predictor variables and the response values of the sample; they learn from what has happened to subjects in the sample in order to predict new subjects. Here is a selected list of supervised methods that can be used in the process of building a prediction model:

1. Stepwise Elimination [18]
2. Support Vector Machines [16]
3. Bump Hunting [19]

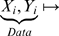
 LASSO and Lars [17] 
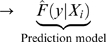

5. Random Forests [20]

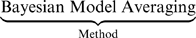
 [21]

Note that the “methods” listed in the previous display are general strategies that do not directly yield a prediction model. In practice it is often necessary to adapt and extend a particular method and to combine it with a dimension reduction step, such as a principal component analysis, or a missing value imputation step. The choice of available methods also depends on the type of the response variable, i.e. whether it is a continuous, binary, or right censored event time variable.

### Playing

From the full data set 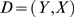 a referee, who may be one of the players, generates 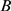 bootstrap samples 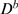, 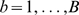 either by sampling of individuals without replacement (subsampling), or with replacement (resampling).

Each player applies the chosen strategy to each of the bootstrap samples and builds prediction models 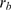, where 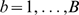, for predicting the conditional probability distribution function of the response variables given the predictor matrices of the bootstrap samples:
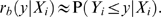
(1)


Here 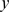 runs through the range of the response variable and the model can be applied to the predictor values of any new subject from the same population. For example, if the response is binary, with classes 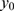 and 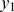, then 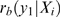 is the predicted risk for a subject with predictor values 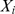 to be in class 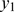. Each player also applies the chosen strategy to the full data set and the resulting prediction model is called the full model and denoted 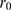 in what follows.

### Rules

1. Each player reveals the chosen strategy by referring to original publications of the method and by accurately documenting all modeling steps.
2. Each player repeats all data dependent modeling steps in each bootstrap sample. The steps may not depend on the full data in any way. A corresponding computer program has to be made available to the other players.
3. The model performance is evaluated by the referee with a strictly proper scoring rule (see the next section).

Apart from these requirements, it is explicitly wanted that the strategies are optimized, tuned, boosted, etc., with respect to the predictive performance of the resulting model.

### Evaluation

A strictly proper scoring rule is chosen to assess the predictive performance. A scoring rule 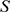 assigns a real valued score 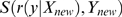 to a new subject with response 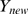 for which the model 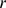 predicts the probability distribution 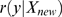. We may assume without loss of generality that a lower score indicates better predictive performance of the model. A scoring rule is called strictly proper if the true conditional probability distribution 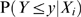 is the unique optimizer [22]. Standard choices are the logarithmic score and the Brier score for binary response variables [9] and the continuous rank probability score for continuous response variables [10]. A time-dependent version of the Brier score and the continuous rank probability score can be used for right censored event time responses [23].

The continuous rank probability score corresponds to the integral of the Brier scores for the associated binary probabilistic predictions at all real-valued thresholds [24]; it is given by
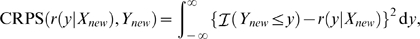
(2)where 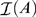 is the indicator function for the event 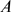. The continuous rank probability score penalizes predictions less severely when their probabilities are close to the true outcome, and more severely when their probabilities are farther from the actual outcome. In practice the integral in the last display can be approximated by a sum over a grid 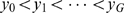 where 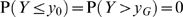:
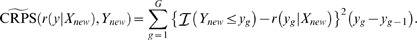
(3)


For all players the scoring rule is applied to evaluate the models fitted in the bootstrap samples. The subjects not in the 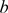
th bootstrap sample are called out-of-bag. They are “new” subjects for the prediction models build with the data of the 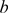
th bootstrap sample, and this is utilized in the bootstrap cross-validation estimate of the generalization performance (GP):
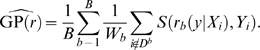
(4)Here 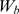 is the number of the subjects not in the 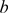
th bootstrap sample. The player whose strategy optimizes the generalization performance wins the game and the corresponding full model is the winning model.

### Benchmarks

Proper benchmarks are important for the interpretation of model performance [15]. Here we use the apparent performance of each strategy which is the performance of the full model when it is evaluated in the full data:
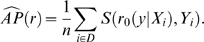
(5)


This yields an upper bound for the generalization performance of the prediction model 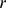, since it is easier to predict the subjects that have been used to build the model. A lower bound is the performance of a strategy that ignores all predictors (null model). If the response variable is binary then the null model predicts the estimated prevalence to every subject. If the response is continuous then the empirical distribution function yields a null model and for a right censored event time the Kaplan-Meier estimate plays this role.

## Application

### VAML: Material

The Nugenob study is a European multi-center study, whose main objective is to explore the role of interactions between macro-nutrient composition of the diet and specific genetic variants [25]. From the original Nugenob cohort comprising 750 European Caucasians, available for our study were the metabolomic profiles from 99 individuals. The fat oxidation capacity was measured for these individuals as the respiratory quotient, i.e. the ratio between the carbon dioxide production and oxygen consumption. Metabolomic profiling was based on plasma samples using 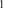
H-NMR and LC-MS techniques. See [26] for information on subject selection, subject characteristics and details on the metabolomic profiling.

In order to predict the respiratory quotient, the players of the VAML game were given 7599 spectral variables from the 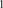
H-NMR, 922 variables from LC-MS metabolic profiles, and the conventional factors age, body weight, body height, and waist circumference. The data used in the game corresponds to 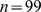 subjects, 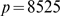 predictor variables and the respiratory quotient response.

### VAML: Aim

The aim was to predict the conditional probability distribution of the respiratory quotient given the predictor variables.

### VAML: Playing

**Figure 1:**
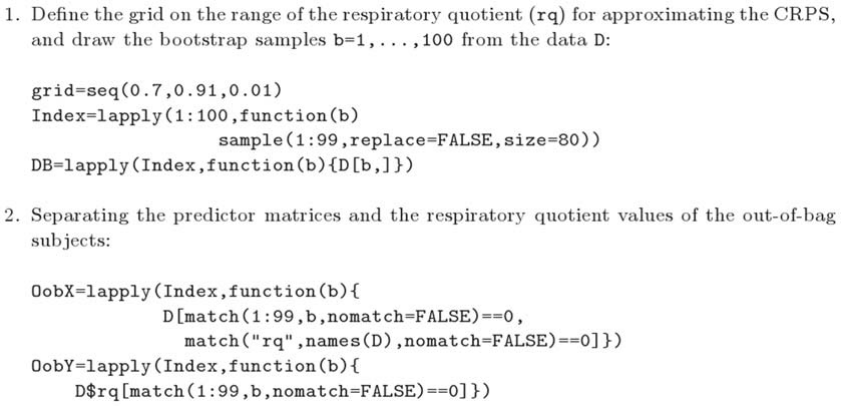
Game setup in R. Extracts from the R script used for setting up the VAML Nugenob game.

TAG was elected as the referee. He sampled 100 bootstrap subsamples of size 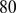 (without replacement) from the 99 subjects (Figure 1). Each player received the 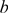
th bootstrap subsample and the predictor matrix of the 19 subjects not sampled in the 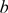
th bootstrap subsample. The observed respiratory quotient values of the 99 subjects ranged between 0.71 and 0.91.

### VAML: Strategies

#### Author THP: Random forest

**Figure 2:**
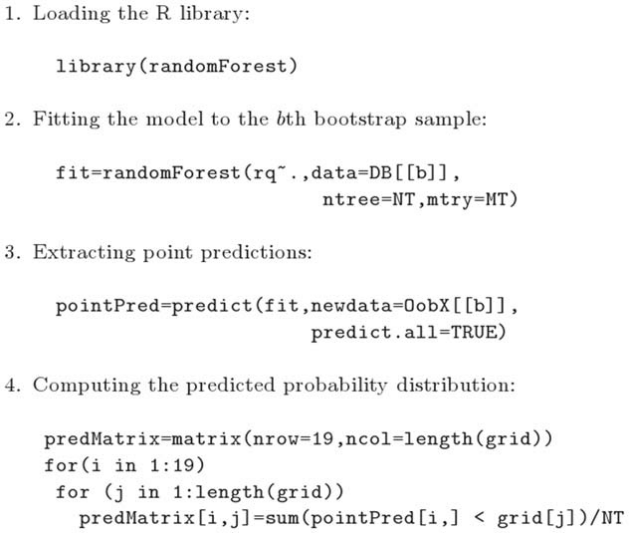
Random forest model. Extracts from the R script that THP used for building the random forest model. The number of trees (NT) and the number of variables tried at each split (MT) are obtained as described in the text.

A random forest model [4] is a classifier which predicts the response based on a majority vote of an ensemble of decision trees [27]. Possible tuning parameters of a random forest model are the number of decision trees and the number of variables used in the split at each internal node of the tree. THP selected these parameters, separately for each of the 100 bootstrap samples, which minimized the 10-fold cross-validated continuous rank probability score: the optimal number of decision trees was searched in the set 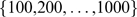; the optimal number of variables tried at each split was searched in the set 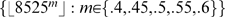. The predicted probability distribution of the respiratory quotient at threshold 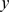 for an out-of-bag subject was computed as the fraction of trees which predicted the respiratory quotient of this subject below 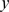 (Figure 2).

#### Author AA: Support vector machines

**Figure 3:**
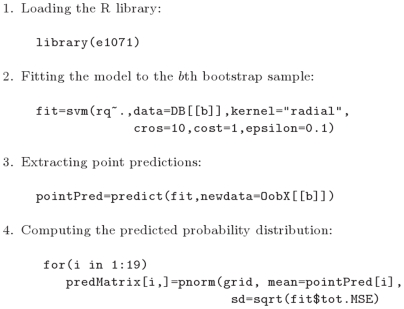
Support vector machine model. Extracts from the R script that AA used for building the support vector machine (SVM) model.

Originally support vector machines [16] were developed for classifying binary outcome. Nowadays, support vector machines have become a popular choice in a wide range of biological applications. Classification is achieved by an affine set that in a given space maximizes a distance between this set and the predictors of both outcome classes. For regression problems and continuous outcome variables one defines a transformation of the predictors into the space using a kernel that takes the predictors and a set of parameters as arguments. The method minimizes the Euclidean norm of the parameters subject to the prediction error being less than 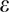 plus some function of a cost parameter. Both the cost parameter and the constant 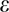 are tuning parameters of the method. AA used the *radial* kernel and used the values 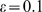 and 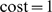 in all bootstrap samples. The probability distribution of the respiratory quotient of the out-of-bag subjects was predicted by a normal distribution with mean equal to the respective point prediction of the respiratory quotients from the support vector machine model. The variance of the predicted distribution was estimated with 10-fold cross-validation for each of the bootstrap samples (Figure 3).

#### Author TAG: LASSO

**Figure 4:**
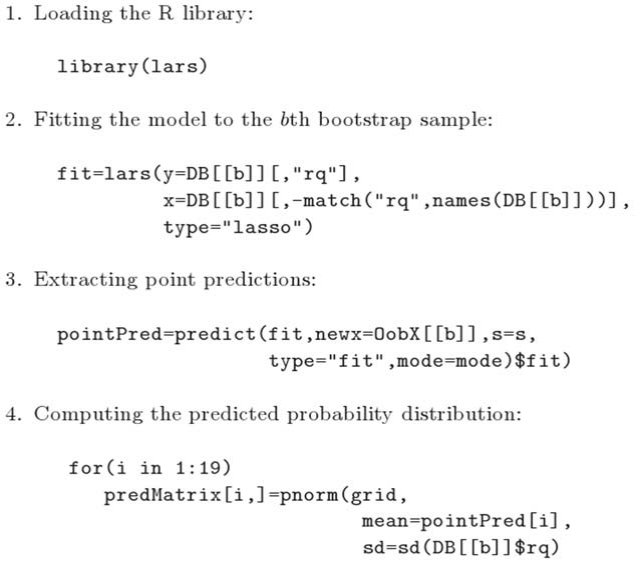
LASSO model. Extracts from the R script that TAG used for building the LASSO model. The shrinkage parameter s is obtained as described in the text.

Least angle regression selects predictors and simultaneously shrinks the regression coefficients by penalization of the likelihood [17]. TAG applied a version of the algorithm with “LASSO option” which provides the entire LASSO path solution of regression coefficients [28]. To select a prediction model from the solution path, TAG repeated 10-fold cross-validation 100 times in each bootstrap sample and used the mean shrinkage of the 100 cross-validation results. The probability distribution of the respiratory quotient of the out-of-bag subjects was predicted by a normal distribution with mean equal to the respective point prediction of the respiratory quotients from the LASSO model. The standard deviation of the respiratory quotient in the 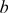
th bootstrap sample was used to estimate the variance of the predicted distribution of the out-of-bag subjects in the 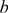
th step (Figure 4).

### VAML: Evaluation

To approximate the continuous rank probability score via formula (3) we used an equidistant grid of 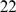 values between 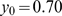 and 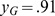 of width 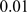. To illustrate graphically the results of the 100 bootstrap-cross-validation steps we computed empirical prediction error curves (PEC) using the formula 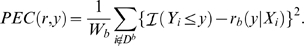
(6)


**Figure 5:**
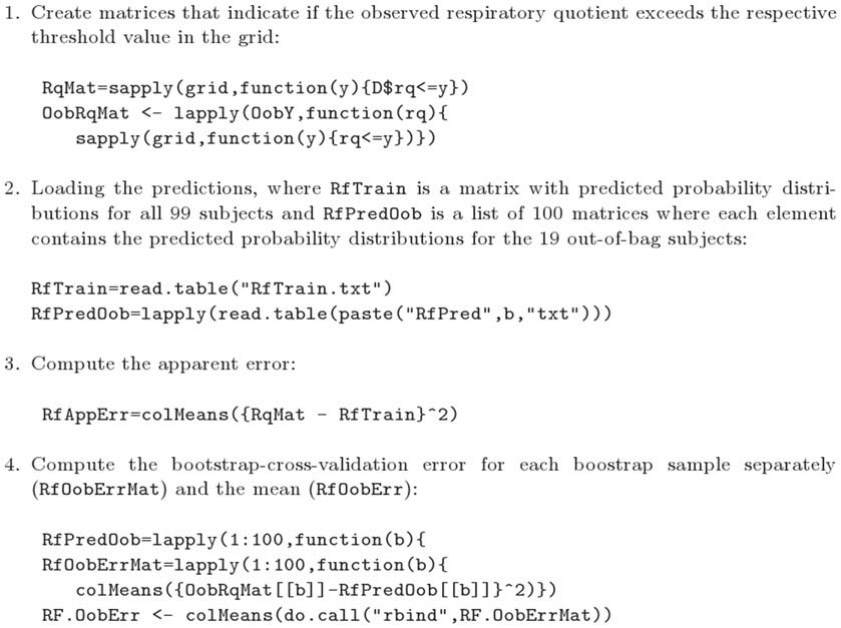
Model evaluation. Extracts from the R script used for evaluating the random forest model in the VAML Nugenob game. The elements of the list RfPredOob are obtained as described in Figure 2. The other two strategies are evaluated similarly.

The estimated continuous rank probability score is the area under the curve 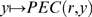, see Figure 5.

**Figure 6:**
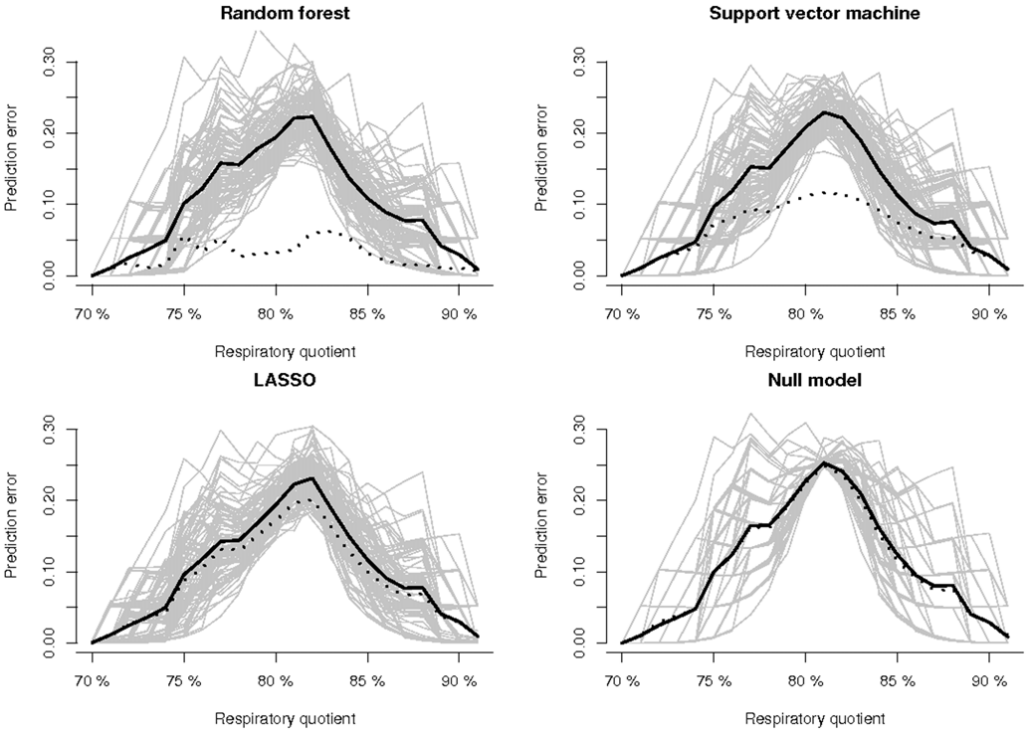
Prediction error curves. Performance of the three strategies and the null model. The gray lines represent the performances of the respective prediction model estimated in the 100 bootstrap cross-validation steps. The solid lines represent the mean bootstrap cross-validation performance and the dashed lines represent the apparent performance.

The pointwise mean of the 100 prediction error curves obtained from the 100 bootstrap-cross-validation steps yields the bootstrap cross-validation estimate of the prediction error curve. The area under this curve is the bootstrap cross-validation estimate of the generalization performance (Table 1). It is well-known that due to the potential of over-fitting, the apparent performance (5) should not be used to compare models. Interestingly, the three modeling strategies yielded quite different apparent error rates: The random forest model showed almost zero apparent error, for the SVM model the apparent error was slightly higher but still very different from the bootstrap cross-validation error, and for the LASSO model exhibited almost no difference between the apparent error and the bootstrap cross-validation error (Figure 6 and Table 1).

**Table 1:** Results of the VAML Nugenob game.

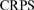	Null model	Random forest	SVM	LASSO
Bootstrap cross-validation error	10.989	10.098	10.173	10.099
Apparent error	10.742	2.776	6.362	8.978

Continuous rank probability scores for the three strategies and the null model that ignores all predictors. The bootstrap cross-validation error is based on 100 bootstrap subsamples of size 80 drawn without replacement from the 99 subjects.

All three models resulted in only slightly lower prediction performance than the benchmark model which ignored the 8525 predictors (Table 1). The random forest model resulted in a lower bootstrap cross-validation error than both the LASSO and SVM method. The LASSO method performed slightly worse than the random forests method, but better than the SVM method. In summary, tuning of the random forest method led to the best prediction model for the respiratory quotient, and hence THP won the game.

### Implementation

All programming was done in R [29]. The random forest, support vector machine, and LASSO models were fitted with the R-libraries randomForest [30], and e1071 [31] and lars [32], respectively.

## Discussion

This article presents a game for comparing statistical strategies for building prediction models. It can for example be applied in a situation where many different strategies are available but neither common knowledge nor theoretical results can immediately advice a solution. Our application of the game to the data of the Nugenob study yields a fair comparison of three quite different approaches, where all of them have previously been successfully applied to address similar problems with relatively many predictor variables and relatively few subjects [33]–[35].

Hand [3] notes: “It may be possible for an expert to tune method A to achieve results superior to method B, but what we really want to know is whether someone untutored in the niceties of method A can do this. Or does method B, presented as a black box and requiring no tuning, generally outperform an untuned method A?”. A VAML game can be used to compare strategies that depend not only on the chosen method but also on the skills of the player.

The game can also be used to test and compare a newly developed algorithm against alternative strategies, where otherwise often the alternative strategies are applied without proper tuning in order to not spoil the importance of the new method. Besides answering the given scientific question, a VAML game leads to enhanced transparency of the method selection step and better didactic reasoning. For example, the game could be used to convince a less experienced researcher, who may or may not have training and experience with statistical analyzes, to choose method B in favor of method A. If the game is played with researchers that have their background and experience in different areas of data analysis, then, as a side effect, the game provides an good opportunity to learn the strategies from each other.

The game is specifically designed for high-dimensional settings were for example many new biomarkers have been measured which potentially could improve individual predictions. Such high-dimensional subject specific information is for example obtained in metabolomics, transcriptomics and with imaging technology, where typically the measurements for a single subject are time and cost expensive. A sensitive strategy is thus crucial for building a prediction model which avoids over-fitting and leads to reproducible results. Without proper validation it may happen that the predictors included in the model are only important for predicting the subjects in the data used for building the model and predicts the outcome of new subjects worse than a null model which ignores all the subject specific measurements [36]. The result of a VAML game is a validated prediction model which outperformed other models and for which the overall benefit of using the predictor information has been quantified using cross-validation and by comparison to a benchmark model which ignores the predictor variables.

To compare different prediction models their performance has to be estimated based on the same data that is available for building the models. The bootstrap-cross-validation approach used here seems appropriate for comparing models, but it has a negative bias and yields pessimistic results regarding the performances of the full models. This happens because a bootstrap sample contains less information than the full data. More advanced resampling approaches like the .632+ estimator [14], [36], [37], which is a smart linear combination of the apparent performance and the bootstrap-cross-validation performance, could potentially reduce this bias. However, for our application we decided not to rely on the .632+ method in view of lacking theoretical arguments regarding its consistency, and since we observed large differences of the apparent performances in our example (Random forest = 2.776, SVM = 6.362, LASSO = 8.978).

We have used bootstrap subsampling where subjects are drawn without replacement from the pool of all patients. This is in agreement with work by Binder and Schumacher [38] who investigated a complexity bias in high-dimensional settings, and also with theoretical results [39] which show that subsampling is more generally applicable than resampling. We have used subsamples of 80 subjects, but it is unclear if this is an appropriate size. Further research is needed to guide the appropriate size of the subsamples for estimating the generalization performance of prediction models. Similarly, the only reason for the number of bootstrap samples used in our application (B = 100) was the computational burden. Further research is needed to get advice and practical rules for finding the appropriate number of cross-validation steps.
